# Quantum signatures of a molecular nanomagnet in direct magnetocaloric measurements

**DOI:** 10.1038/ncomms6321

**Published:** 2014-10-22

**Authors:** Joseph W. Sharples, David Collison, Eric J. L. McInnes, Jürgen Schnack, Elias Palacios, Marco Evangelisti

**Affiliations:** 1 School of Chemistry and Photon Science Institute, The University of Manchester, Manchester M13 9PL, UK; 2 Faculty of Physics, Bielefeld University, PO box 100131, D-33501 Bielefeld, Germany; 3 Departamento de Física de la Materia Condensada and Instituto de Ciencia de Materiales de Aragón (ICMA), CSIC—Universidad de Zaragoza, Pedro Cerbuna 12, 50009 Zaragoza, Spain

## Abstract

Geometric spin frustration in low-dimensional materials, such as the two-dimensional kagome or triangular antiferromagnetic nets, can significantly enhance the change of the magnetic entropy and adiabatic temperature following a change in the applied magnetic field, that is, the magnetocaloric effect. In principle, an equivalent outcome should also be observable in certain high-symmetry zero-dimensional, that is, molecular, structures with frustrated topologies. Here we report experimental realization of this in a heptametallic gadolinium molecule. Adiabatic demagnetization experiments reach ~200 mK, the first sub-Kelvin cooling with any molecular nanomagnet, and reveal isentropes (the constant entropy paths followed in the temperature-field plane) with a rich structure. The latter is shown to be a direct manifestation of the trigonal antiferromagnetic net structure, allowing study of frustration-enhanced magnetocaloric effects in a finite system.

## Abstract (web summary)

The magnetocaloric effect is well understood in spin-frustrated low-dimensional systems, and should be observable in certain high-symmetry molecular structures. Here, the authors report the experimental observation of sub-Kelvin cooling with a molecular magnet, and probe the low-temperature spin behaviour.

## Graphical abstract

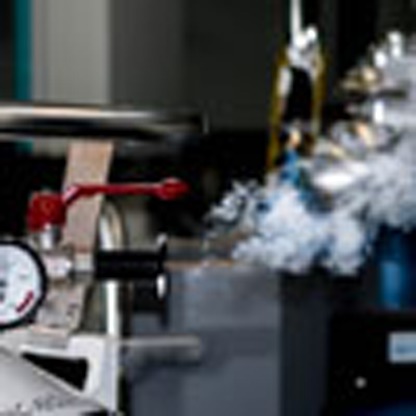

Sub-Kelvin temperatures can be achieved via adiabatic demagnetization of paramagnetic salts^1^,^2^. The underlying physics is the magnetocaloric effect (MCE) that can be evaluated by considering the adiabatic temperature change, which is when the system is driven on a constant entropy (*S*) curve (an isentrope):


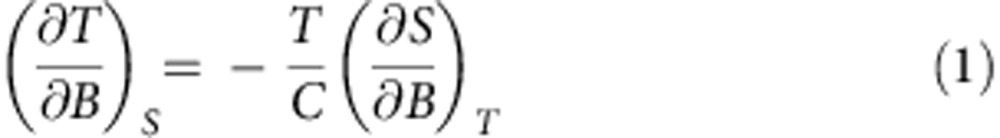


where *C* is the heat capacity, *T* is the temperature and *B* is the applied magnetic field. For a paramagnet, the isentropes are straight lines in a *T*–*B* plane that run through the origin. Interacting spin systems can show a much richer response to magnetic fields and thus very different isentropes. Importantly, the cooling rate can massively outperform those of paramagnets in certain regions of the *T*–*B* plane^3^. The simplest illustration is an antiferromagnetically coupled dimer of *s*=1/2 spin (Fig. 1) where extremes in the cooling rates (even changing sign) are found at the field-induced level crossing between singlet and triplet because the density of states (and hence the low-temperature entropy) peaks at this field.

Such a crossing belongs to the broader class of quantum phase transitions where the ground-state characteristics of a system change (for example, non-magnetic to magnetic, or from gapped to gapless) as a function of an external parameter such as magnetic field, pressure or doping^4^. For MCE, the drastic changes in entropy across a field-induced quantum critical point can give very efficient low-temperature magnetic cooling as recently demonstrated experimentally for a one-dimensional (1D) antiferromagnetic (AF) *s*=1/2 chain^3^. Geometric spin frustration can also give rise to regions of high density of states (and zero-temperature entropy), hence very high cooling rates should also be achievable, for example, when sweeping across the saturation field in such materials. The combination of these features in low-dimensional frustrated magnetic materials, for example, the famous 2D kagome or triangular AF lattices or the 1D saw-tooth AF chain^5^,^6^,^7^,^8^,^9^, makes them attractive targets for enhanced MCE and low-temperature refrigeration. In fact, such effects should be also observable in certain 0D systems, that is, molecular clusters of spins in frustrated geometries^10^,^11^,^12^,^13^. These are a subset of the broader class of molecules known as molecular nanomagnets.

The molecular cluster [Gd_7_(OH)_6_(thmeH_2_)_5_(thmeH)(tpa)_6_(MeCN)_2_](NO_3_)_2_ (‘Gd_7_’; H_3_thme=tris(hydroxymethyl)ethane; Htpa=triphenylacetic acid) consists of a planar centred hexagon of weakly AF-coupled Gd(III) ions (Fig. 2; ref. 14), each of which has an electronic spin *s*=7/2. Hence, this topology is a finite ‘cutout’ of the 2D triangular AF lattice (Fig. 2). Here we model all the magnetic observables of Gd_7_, including sub-Kelvin susceptibility and heat capacity data. We then use this model to calculate the isentropes for Gd_7_, revealing detailed structure in the *T*–*B* landscape due to the frustration. Finally, we follow these isentropes experimentally by direct measurement of the temperature in applied magnetic field cycles under quasi-adiabatic conditions. The experimental data, reproduced by theoretical modelling, show the characteristics of frustration-enhanced MCE; moreover, we achieve cooling to ~200 mK—the first time sub-Kelvin cooling has been achieved with a molecular nanomagnet.

## Results

### Magnetic properties

**Figure 3:**
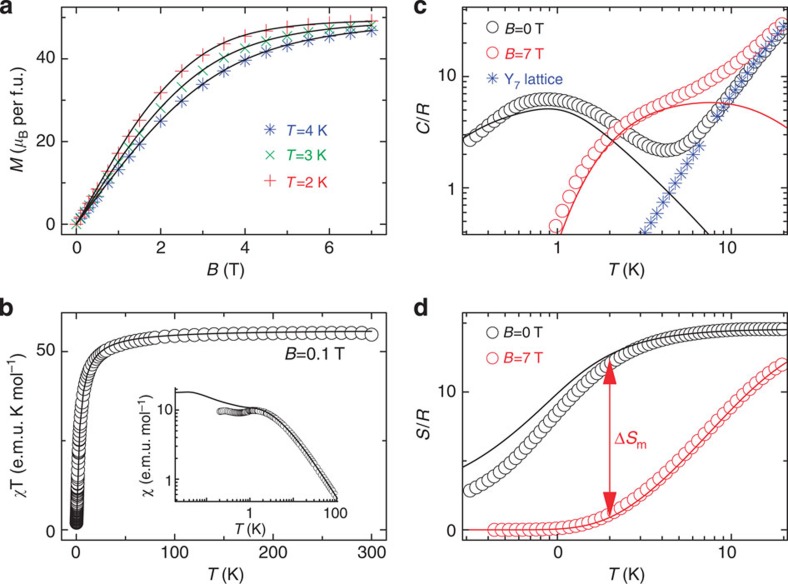
Magnetic properties of Gd_7_. (**a**) Magnetization (*M*) as a function of applied magnetic field (*B*) and temperature (*T*=2, 3, 4 K), and fits (solid lines) from spin Hamiltonian (2). (**b**) Molar magnetic susceptibility (*χ*), in the form of *χT* and *χ* (inset), as a function of temperature, measured in an applied field of 0.1 T, and fits (solid lines). (**c**) Molar heat capacity (*C*) of Gd_7_ as a function of temperature at *B*=0 (black symbols) and 7 T (red), and for its diamagnetic analogue Y_7_ (blue) in nil field giving the lattice (non-magnetic) contribution to *C*. Solid lines are the calculated magnetic contributions to *C*(*B*,*T*) from Hamiltonian (2). (**d**) Magnetic molar entropy, as obtained from *C*(*T*) data for *B*=0 (black) and 7 T (red). Solid lines are the calculated entropies from Hamiltonian (2). Arrows denote the magnetic entropy change, Δ*S*_m_ (see Supplementary Fig. 1). The small deviations between theory and experiment seen in *χ* and *C* at very low temperatures indicate the onset of magnetic dipolar interactions.

Low-temperature magnetic data of Gd_7_ are summarized in Fig. 3. The magnetization (*M*) saturates to the maximum possible 49/2 *gμ*_B_ (where *g* is the electronic *g*-factor) per molecule at 2 K, showing that the full magnetic entropy is accessible (Fig. 3a). The *χT* product, where *χ* is the molar magnetic susceptibility, has the value calculated for non-interacting Gd(III) ions at room temperature (56.2 e.m.u. K mol^−1^) and decreases only slowly on cooling down to ~50 K before decreasing rapidly on further cooling (Fig. 3b), denoting a dominant AF interaction. That Gd_7_ has a richer physics than a simple paramagnet is manifested in the very-low-temperature susceptibility, which goes through two shallow maxima, at 1–2 K and at 0.2–0.3 K (Fig. 3b, inset). Above 4 K, the molar heat capacity (*C*) in zero applied field is dominated by lattice phonon modes of the crystal, that is, non-magnetic contributions (Fig. 3c). This is confirmed from *C*(*T*) data on the isostructural and diamagnetic yttrium analogue [Y_7_(OH)_6_(thmeH_2_)_5_(thmeH)(tpa)_6_(MeCN)_2_](NO_3_)_2_ (‘Y_7_’), which overlay those of Gd_7_ at higher temperatures. The phonon heat capacity can be described by the Debye model, which simplifies to a *C*/*R*=*aT*^3^ dependence (*R* is the gas constant), where *a*=1.35 × 10^−2^ K^−3^ for Gd_7_ and Y_7_, at the lowest temperatures. The magnetic contribution to the *C*(*T*) data for Gd_7_ consists of a broad hump that shifts to higher temperature on increasing the applied magnetic field (Fig. 3c).

### Magnetic modelling

We have modelled all these magnetic data assuming the simple Heisenberg spin Hamiltonian:


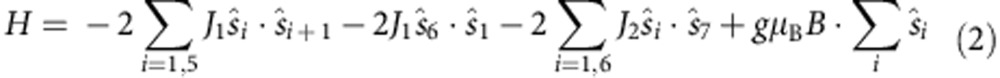


where *J*_1_ is the exchange interactions between nearest neighbours on the hexagon (spins 1–6), and *J*_2_ is the interactions between each of these spins and the central Gd (spin 7). The huge matrix dimension of 8^7^ requires exploiting group theoretical methods^15^,^16^ (and the approximate C_6_ molecular symmetry) for full matrix diagonalization. We find *J*_1_=−0.090(5) K, and *J*_2_=−0.080(5) K with *g*=2.02 reproduces all the experimental magnetic observables (Fig. 3). Only at the very lowest temperatures, the weak-field susceptibility and zero-field heat capacity show slight deviations between calculated and experimental data. For instance, the calculated susceptibility reproduces the shallow two-peak structure, with the higher-temperature feature agreeing well but the lower temperature one calculated to be at ~0.05 K rather than the experimental 0.2–0.3 K. Most likely, these discrepancies are due to weak magnetic dipolar interactions, which are not incorporated in the theoretical model. Dipolar interactions modify the structure of energy levels and can determine (on the mean-field level) an internal field; both become relevant in proximity of absolute zero and zero applied field.

### Experimental evaluation of the MCE

**Figure 4:**
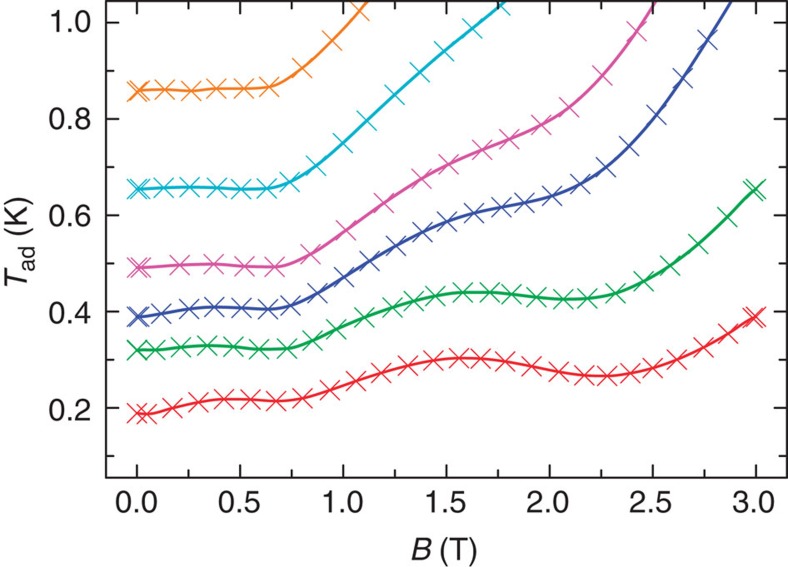
Experimental temperature evolution of Gd_7_ on applied field changes. The different curves (which correspond to isentropes) are for different initial temperature and applied field conditions *T*_0_ and *B*_0_, respectively; solid lines are guides to the eye. The magnetic entropy values are *S*/*R*=1.6, 2.9, 3.5, 4.4, 5.9 and 7.6, from bottom to top, respectively. Data are shown for the sub-Kelvin temperature regime (see Supplementary Fig. 4 for a wider temperature range).

The MCE can be evaluated indirectly for a given applied field change from the experimental *C*(*B*,*T*) (for example, Fig. 3d) and *M*(*B,T*) data via Maxwell’s relations^17^: values for Gd_7_ derived from these two observables are in very good agreement (Supplementary Fig. 1). Here we have also performed direct experimental measurements of the MCE for continuous field variations, that is, the temperature evolution via magnetization–demagnetization cycles that we perform under controlled quasi-adiabatic conditions, using the set-up and protocols described in Supplementary Note 1 and ref. 18. Supplementary Fig. 2 displays a representative full magnetic field cycle, and Supplementary Fig. 3 a representative demagnetization process from an initial temperature *T*_0_=0.50 K and field *B*_0_=2 T. We show both the raw temperature data and those for an ideal adiabatic process, that is, corrected for unavoidable thermal losses (non-adiabaticity) that have been evaluated independently (see Supplementary Note 1). By this method, we experimentally follow isentropes in the *T*–*B* plane for different *B*_0_ and *T*_0_ (up to 3 T and 3 K, respectively; Fig. 4; Supplementary Fig. 4). The general trend is a decrease in *T* as *B* is decreased, as expected. There are two important results from these adiabatic demagnetization experiments. First, we achieve temperatures as low as *~*200 mK. Despite many indirect MCE studies on molecular nanomagnets, this is the first direct experimental demonstration of sub-Kelvin cooling with such a species. Second, in contrast to the straight-line isentropes found for simple paramagnets, a rich structure is observed.

On demagnetization from *B*_0_=3 T, a minimum (at 2.2 T) is found in the isentropes, that is, the sample cools rapidly (large positive slope) then heats (negative slope), strongly reminiscent of the behaviour observed recently for a 1D AF chain at a quantum critical point^3^. On decreasing the field further, the *T*(*B*) curves go through a second minimum (at ~0.7 T). As far as we are aware, such multiple peak behaviour has not been observed previously. However, secondary minima have been predicted theoretically for ideal frustrated 2D lattices as a function of decreasing size^5^,^7^, and also for very high-symmetry (cuboctahedral, icosidodecahedral) frustrated clusters^10^,^11^,^12^,^13^, that is, they arise as a function of finite-size effects.

### Comparison with calculated results

**Figure 5:**
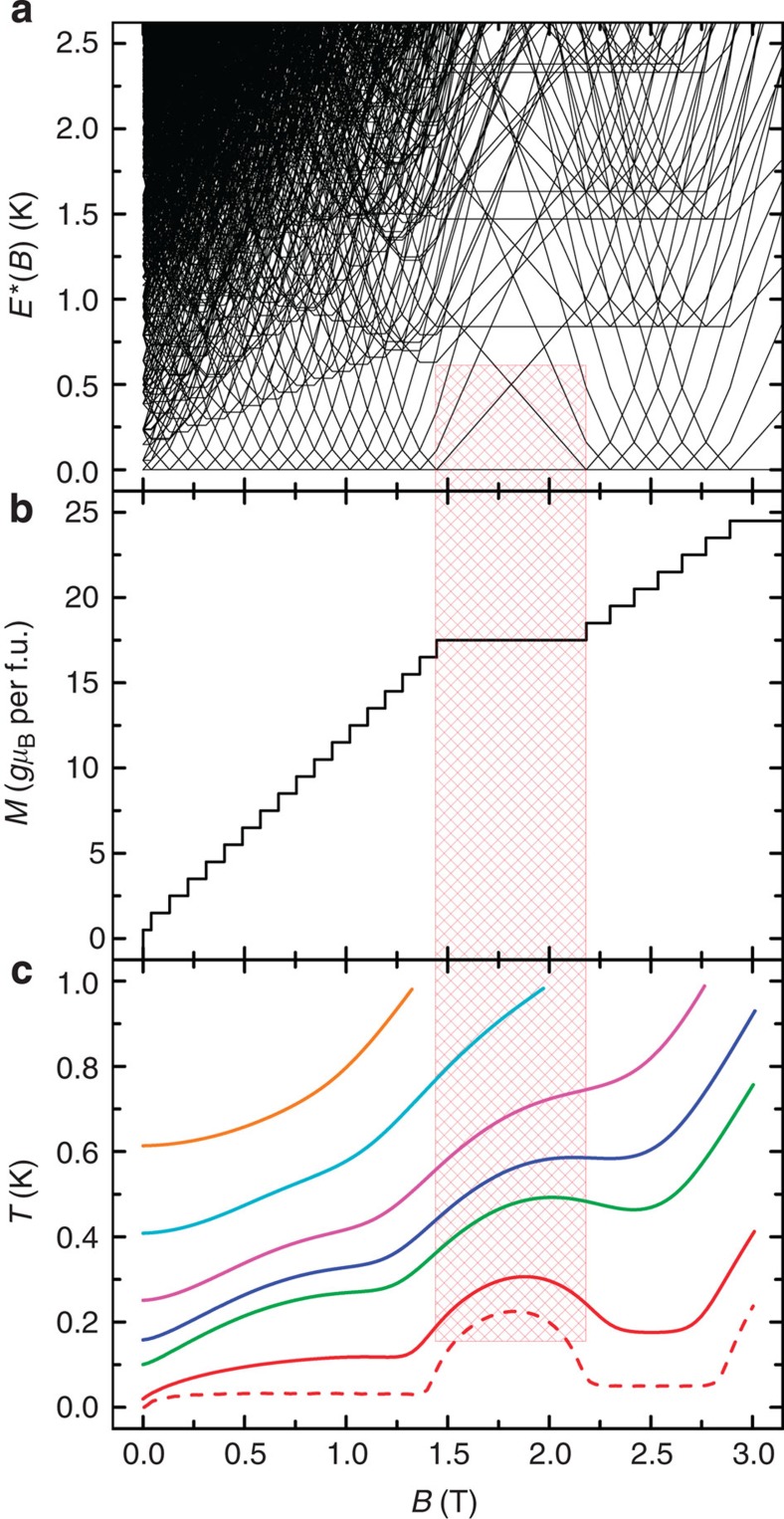
Calculated level structure and isentropes for Gd_7_. (**a**) Zeeman diagram for Gd_7_ calculated from spin Hamiltonian (2), shown as excitation energies (E*=*E*_i_−*E*_0_; where *E*_i_ and *E*_0_ are the energies of the ith and ground Zeeman states, respectively, at a given *B*) as a function of applied field, highlighting the varying low-lying density of states. (**b**) Calculated zero-Kelvin *M*(*B*) curve. The plateau at *M*=35/2 *gμ*_B_ between *B*=1.4 and 2.2 T coincides with the region of low density of states in **a**; saturation (at 49/2 *gμ*_B_) is achieved at *B*=2.9 T. (**c**) Theoretically calculated isentropes for magnetic entropy values *S*/*R*=1.0, 1.6, 2.9, 3.5, 4.4, 5.9 and 7.6, from bottom to top, respectively. The solid lines are those isentropes that match the experimental entropies in Fig. 4 and can be compared with these directly. The red dashed curve shows a further low-entropy isentrope, lower than those experimentally accessible. The shaded red box highlights the magnetic field region giving the low density of states, the magnetization plateau and the maximum in the isentropes.

We have calculated theoretical isentropes from the entropy function *S*(*T*,*B*) based on the parameters from spin Hamiltonian (2) (see Fig. 5c). We have done this for the experimental entropies that belong to the isentropes shown in Fig. 4 to allow a direct comparison, and for a lower entropy to emphasize the shape of the isentropes. The agreement with the experimental curves is remarkable, showing the double minimum in *T*(*B*) and consequent multiple cooling regimes. The agreement becomes poorer for the lowest temperatures and small fields because the aforementioned dipolar interactions become relevant. The latter, which are not included in our model, ultimately limit the base temperature reached by adiabatic demagnetization. Analysing the Zeeman diagram is difficult because of the massive (8^7^) number of levels; in Fig. 5a, we plot the excitation energies (E*=*E*_i_−*E*_0_, where *E*_i_ and *E*_0_ are the energies of the ith and ground Zeeman states, respectively, at that field) to make the changes in density of states in certain field ranges more visible. The zero-temperature saturation field is ~2.9 T (that is, above which the ground state is singly degenerate and the magnetic entropy is nil; Fig. 5b). Below this saturation field, there is a high degeneracy of low-lying states (high entropy), hence rapid magnetic cooling is observed on demagnetizing towards 2.5 T (positive slope isentrope; Fig. 5c). Between about 2.2 and 1.4 T, the density of states is much lower (Fig. 5a), giving a plateau in the zero-temperature magnetization curve (Fig. 5b), hence demagnetizing into this region decreases the entropy and leads to heating (negative slope isentrope; Fig. 5c). Below 1.4 T, the density of states increases again, and we are back in a region of cooling.

## Discussion

Several frustrated antiferromagnets, including 2D kagome and triangular lattices and certain 0D polytopes, have been predicted to show plateaus in their zero-temperature magnetization curves together with regions of lower densities of states^5^,^7^,^10^,^11^,^12^,^13^. The uneven distributions of intervals between ground-state level crossings is a clear signature of frustration^13^, and is the reason for the peaks observed in the isentrope distribution. This frustration arises because *J*_1_≈*J*_2_, and test calculations show that the isentrope peaks are quickly destroyed by smaller values of *J*_2_/*J*_1_ (hence, weakening the frustration; Supplementary Fig. 5).

**Figure 6:**
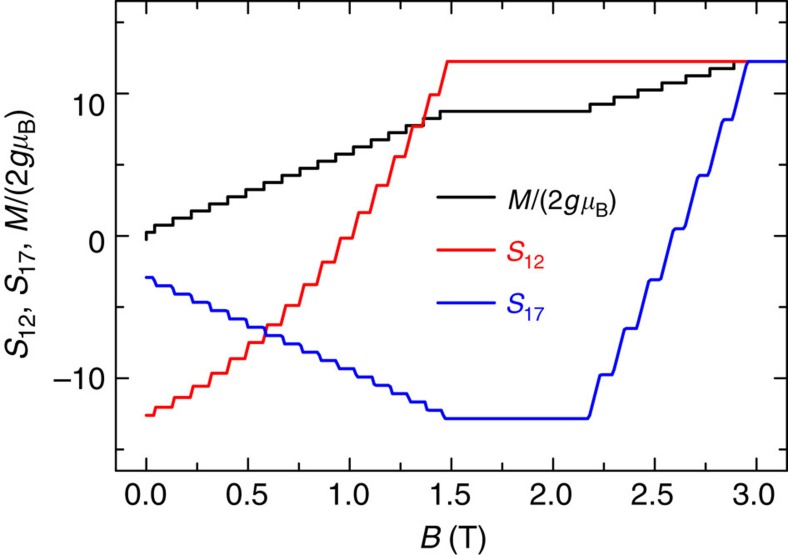
Ground-state nearest-neighbour spin correlation functions. The spin functions 
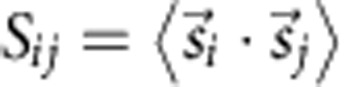
 are calculated as a function of applied magnetic field. *S*_*12*_ measures the correlation of neighbouring spins on the hexagon, while *S*_*17*_ those of a spin on the hexagon and the central spin. |*S*_*ij*_|=49/4 corresponds to full (anti)parallel alignment. The zero-temperature *M*(*B*) curve (scaled by 2 *gμ*_B_) is included to highlight the coincidence of the magnetization plateau with the spin configuration corresponding to the full antiparallel alignment of the central spin with all the other spins.

Insight into the microscopic origin of the zero-Kelvin magnetization plateau in Gd_7_ is gained from evaluating the ground-state nearest-neighbour spin–spin correlation functions 
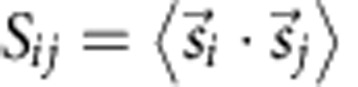
 as a function of the applied field (Fig. 6), evaluated by numerical differentiation of the ground-state energy with respect to *J*_1_ (*S*_*12*_) or *J*_2_ (*S*_*17*_). Calculation from the ground-state eigenfunctions is prohibitive given the enormous Hilbert space. The *S*_*12*_ function, that is, for neighbouring spins on the hexagon, grows from a fully antiparallel alignment (maximum negative *S*_*12*_) at *B*=0 to a saturated parallel alignment (maximum positive *S*_*12*_) at *B*=1.4 T. The *S*_*17*_ function, that is, for a spin on the hexagon correlated with the central spin, starts at a small negative value and becomes more negative with increasing *B*, reaching a fully antiparallel alignment at *B*=1.4 T. *S*_*17*_ is then constant until *B*=2.2 T after which it increases, reaching full parallel alignment at *B*=2.9 T (and saturation of the magnetization at 49/2 *gμ*_B_ per molecule). Hence, the magnetization plateau between 1.4 and 2.2 T corresponds to a region of stability for the spin configuration with all the spins on the hexagon fully aligned parallel with each other but fully antiparallel to the central spin, consistent with the calculated plateau magnetization of 35/2 *gμ*_B_ per molecule (Fig. 5b). In fact, the Gd_7_ structural motif is one of the smallest fragments of the triangular AF net that would be predicted to show such effects. For example, the smallest possible frustrated fragment—an equilateral triangle—has no such ‘meta-stable’ intermediate spin configuration, hence no magnetization plateau and a much simpler isentrope structure (Supplementary Fig. 6).

Many molecular nanomagnets have now been proposed for low-temperature magnetic refrigeration (see, for example, refs 17, 19, 20, 21, 22, 23, 24, 25, 26), even in principle to the single-molecule level^27^, due to the high magnetic degeneracies that can be built in by appropriate choice of metal ion and a favourable exchange coupling scheme. Almost all these studies have relied on indirect MCE measurements from magnetization or heat capacity data, which are analysed to predict some maximum magnetic entropy change for a maximum field change (typically 0–5 T on a conventional SQUID magnetometer) and certain initial temperature. Such indirect analyses can give impressive headline figures but ignore the details of the exchange coupling (other than, for example, being ‘weak’, hence giving large quasi-degeneracies in zero field). Hence, they are blind to the structure and true beauty of the isentropes that are a function of the exchange couplings. Here we have revealed the richness of the isentropes in Gd_7_ via direct MCE studies, including the first experimental achievement of sub-Kelvin cooling with a molecular nanomagnet, with experimental and theoretical results in excellent agreement. Our results show that it is possible to design the cooling power of molecular materials by choosing an appropriate topology of magnetic couplings between the interacting spins, hence exploiting the great control of the latter given by molecular coordination chemistry.

The enhanced MCE we observe in certain regions of the *T*–*B* plane for Gd_7_ also confirms long-standing predictions about unusually large cooling rates in frustrated spin 0D polytopes as well as low-dimensional extended spin lattices^5^,^6^,^7^,^8^,^9^,^10^,^11^,^12^,^13^. Indeed, the Gd_7_ molecule is a cutout of the triangular AF lattice, with imposed geometric spin frustration giving exact or near degeneracies at certain applied magnetic fields, and serves as a finite-size realization of these predictions. Such finite systems are useful in their own right, as demonstrated here, but also enable exact numerical analysis, hence giving insight into the behaviour of infinitely extended systems. If bigger molecular fragments of the triangular AF net could be prepared (such molecules are known for some d-block ions, see refs 28, 29), this would allow fascinating insight into the transition from discrete to bulk behaviour in frustrated systems.

## Methods

### Materials

[Gd_7_(OH)_6_(thmeH_2_)_5_(thmeH)(tpa)_6_(MeCN)_2_](NO_3_)_2_ (‘Gd_7_’) was prepared as reported previously^14^. Its diamagnetic and isostructural analogue [Y_7_(OH)_6_(thmeH_2_)_5_(thmeH)(tpa)_6_(MeCN)_2_](NO_3_)_2_ (‘Y_7_’) was prepared by an identical method but with substitution of the appropriate metal precursor. Solvothermal reaction of Y(NO_3_)_3_·6H_2_O (0.085 g, 0.22 mmol) with H_3_thme (0.11 mmol), Htpa (0.11 mmol) and NEt_3_ (0.165 mmol) in MeCN (8 ml) at 100 °C for 12 h, followed by slow cooling (0.05 °C min^−1^) to room temperature, gave colourless crystals of the product in ~40% yield. The formulation is confirmed by elemental analysis, powder X-ray diffraction (Supplementary Fig. 7) and a single-crystal unit cell determination, which show that Y_7_ is isostructural with Gd_7_. Elemental analysis (%) for Y_7_C_154_H_164_N_4_O_42_ (found:calculated): C 53.26:54.96; H 4.45:4.91; N 1.74:1.66.

### Measurements

Magnetization measurements down to 2 K and heat capacity measurements using the relaxation method down to 0.3 K were carried out on powdered crystalline samples by means of commercial setups for the 0–9 T magnetic field range. Susceptibility measurements were extended down to 0.1 K with a homemade susceptometer, installed in a ^3^He-^4^He dilution refrigerator. Direct MCE measurements were performed on a pressed pellet sample mounted on a sapphire plate attached to a Cernox resistance thermometer, attached by wires to a controlled thermal bath. Each MCE measurement started with the sample at zero applied magnetic field and at temperature *T*_0_, and comprised: (a) gradual application of a magnetic field, up to a maximum *B*_0_; (b) relaxation until the sample reached the thermal equilibrium with the bath; (c) gradual demagnetization down to *B*=0; and (d) relaxation at zero field until the sample reached thermal equilibrium at *T*_0_. During the whole procedure, the temperature *T* and applied magnetic field *B* were recorded continuously. See Supplementary Note 1 for full details.

## Author contributions

J.W.S. made and characterized the materials, under the supervision of D.C. and E.J.L.M. E.P. and M.E. designed and performed the quasi-adiabatic magnetocaloric and magnetic experiments. J.S. modelled the magnetic data. E.J.L.M., J.S. and M.E. wrote the manuscript with further contributions from all authors.

## Additional information

**How to cite this article**: Sharples, J. W. *et al.* Quantum signatures of a molecular nanomagnet in direct magnetocaloric measurements. *Nat. Commun.* 5:5321 doi: 10.1038/ncomms6321 (2014).

## Supplementary Material

Supplementary Information Supplementary Figures 1-7, Supplementary Note 1 and Supplementary References

## Figures and Tables

**Figure 1:**
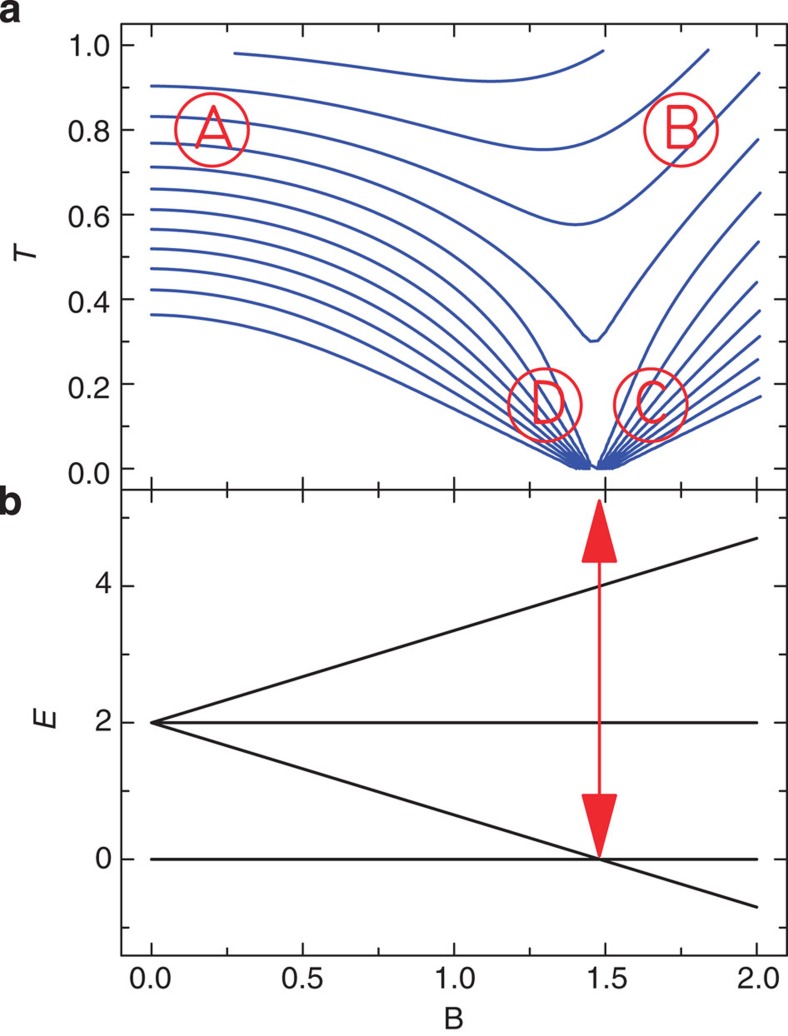
Theoretical isentropes for an antiferromagnetically coupled dimer of *s*=1/2 spins. (**a**) Calculated isentropes in the temperature (*T*)–applied magnetic field (*B*) plane for the dimer with the Zeeman diagram in **b**. The two lowest-energy Zeeman levels cross at a field that depends on the magnitude of the exchange coupling, giving a maximum in the density of states. Simple paramagnets have linear isentropes, always giving a decrease in *T* as *B* is decreased. While the cooling rate for the antiferromagnetically coupled dimer is similar in the high *T*–*B* region (B), in other regions it can be reduced (A), drastically enhanced (C) or even have the opposite sign (D, that is, heating occurs on decreasing the field). In an adiabatic process (a process of constant entropy), the system runs along its present isentrope. The extreme behaviour at (C) and (D) is due to the level crossing and the consequent rapid changes in entropy.

**Figure 2:**
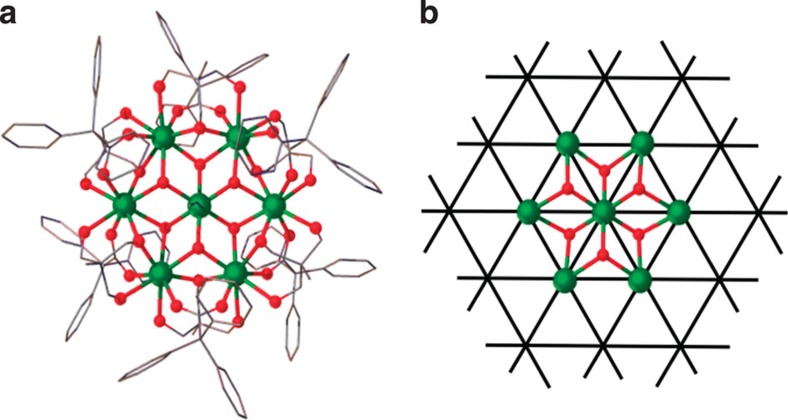
Structure of the complex dication of Gd_7_. Crystal structure (**a**) and the Gd_7_(OH)_6_ core mapped onto an ideal triangular net (**b**), emphasising the relationship of the spin topology with the frustrated triangular AF lattice. Scheme: Gd (green), O (red), N (blue), C (framework), H omitted for clarity.
