# Supplementary material for: Quantum signatures of a molecular nanomagnet in direct magnetocaloric measurements
Source: Nat Commun. 2014 Oct 22;5:5321. doi: 10.1038/ncomms6321 (PMC4220460; doi:10.1038/ncomms6321)
Supplement: Supplementary Information — Supplementary Figures 1-7, Supplementary Note 1 and Supplementary References [file ncomms6321-s1.pdf]

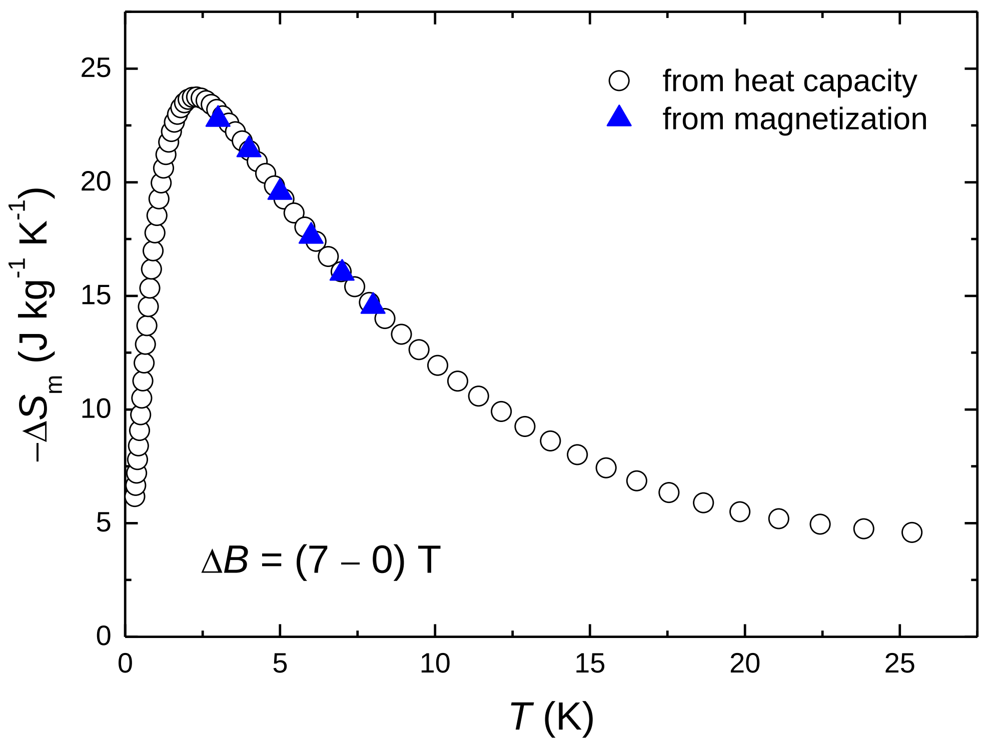


**Supplementary Figure 1**. **Indirect MCE measurements for $Gd_7$.** Changes of the magnetic entropy for $Gd_7$, collected as a function of the starting temperature for an applied field change of 0 – 7 T, determined indirectly from experimental magnetization $M(B,T)$ (blue triangles) and heat capacity $C(B,T)$ (black circles) data, using well-known procedures (see, *e.g.*, Supplementary Reference 1).

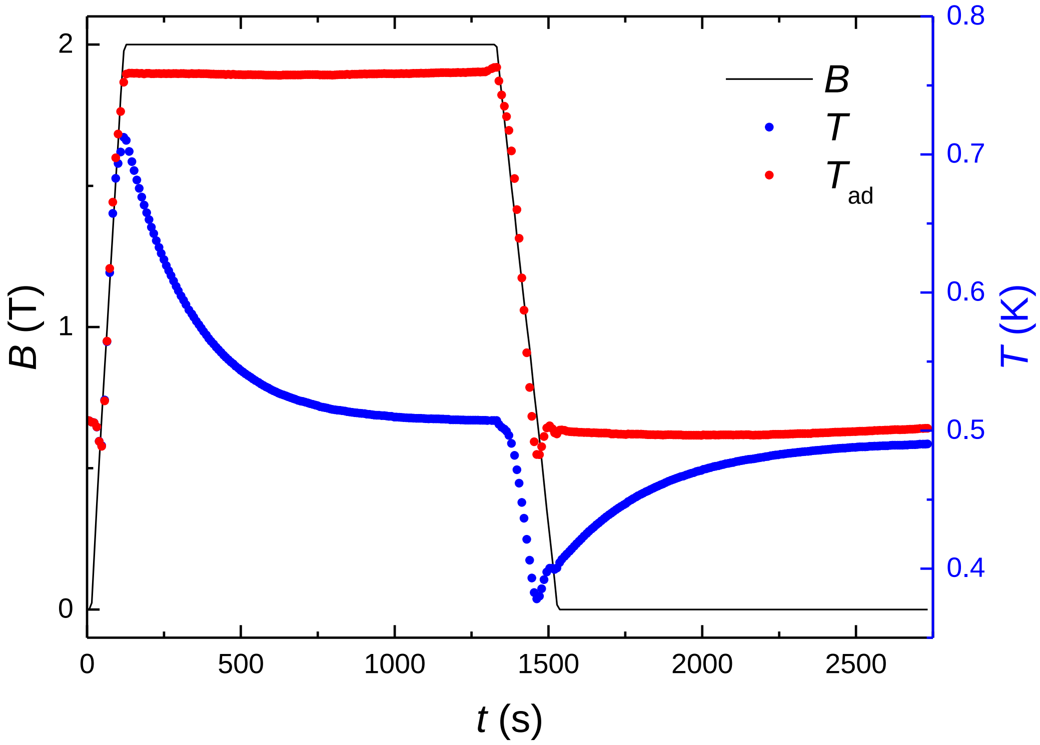


**Supplementary Figure 2**. **Experimental temperature evolution of $Gd_7$ on a representative magnetization-demagnetization cycle.** These data are collected for $T_0$ = 0.50 K and $B_0$ = 2 T: applied magnetic field ($B$; black line), measured temperature ($T$; blue) and corrected for an ideal adiabatic process ($T_{ad}$; red). The temperature and applied field are recorded at time ($t$) intervals approximately every 10 s throughout.

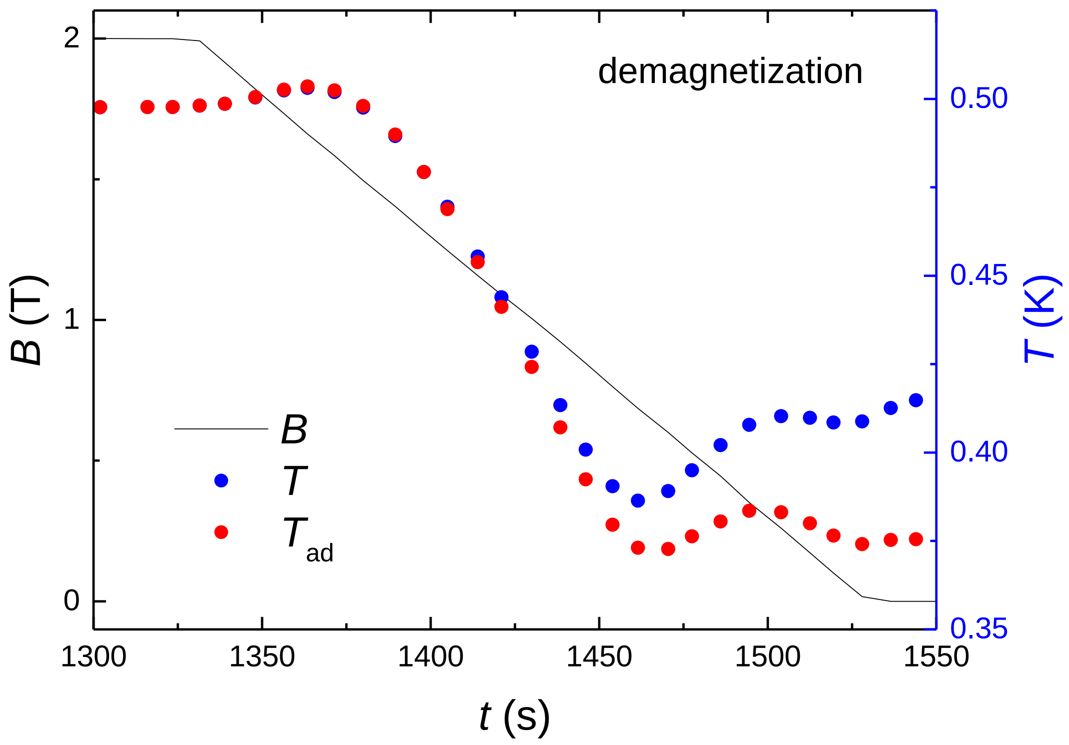


**Supplementary Figure 3**. **Experimental temperature evolution of $Gd_7$ on a representative demagnetization half-cycle.** These data are collected for $T_0$ = 0.50 K and $B_0$ = 2 T: magnetic field (black line), measured temperature, $T$ (blue) and corrected for an ideal adiabatic process, $T_{ad}$ (red). The temperature and applied magnetic field are recorded at time ($t$) intervals approximately every 10 s throughout.

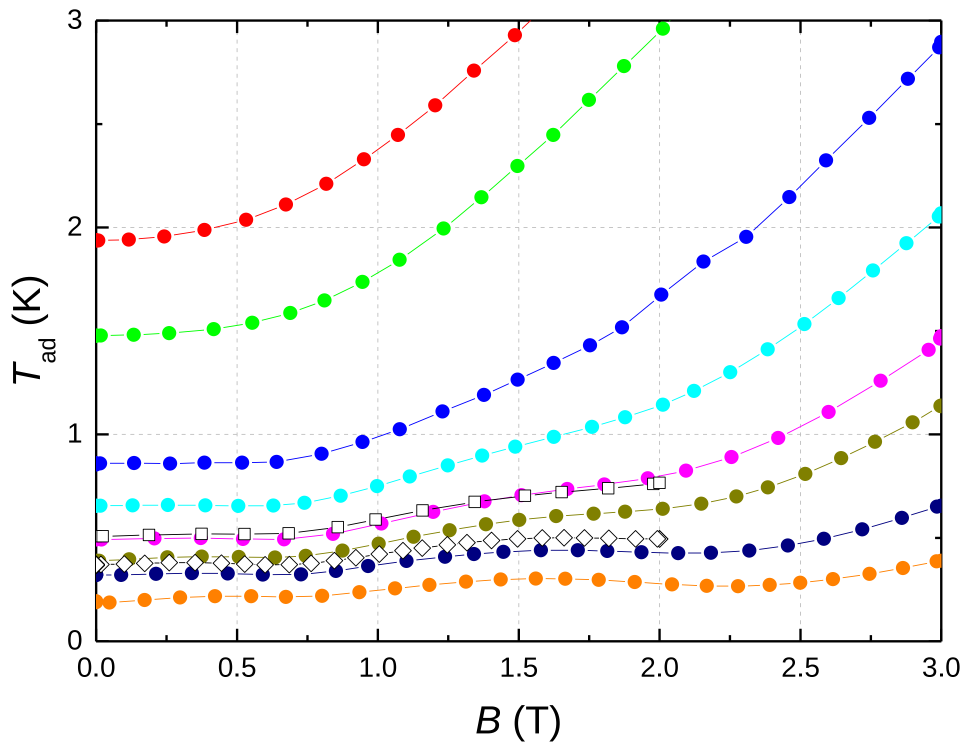


**Supplementary Figure 4**. **Experimental isentropes for $Gd_7$.** These data, collected for $T_0$ up to 3 K and $B_0$ up to 3 T, are corrected for the unavoidable lack of adiabatic conditions. Empty, white symbols are data from Supplementary Figures 2 and 3 on magnetization (square symbols) and demagnetization (rhomb symbols). Lines are guides to the eye.

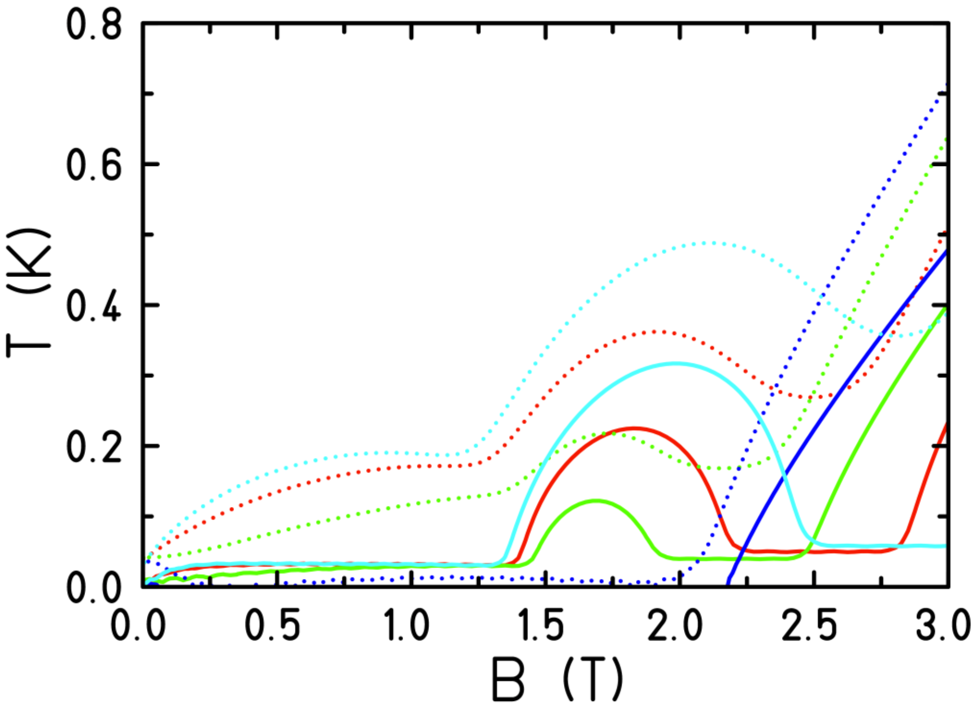


**Supplementary Figure 5**. **Theoretically calculated isentropes for $Gd_7$ with fixed $J_1$ and varying $J_2$.** These curves are obtained for $J_1 = -0.09$ K and $J_2 = -0.09$ (cyan), $-0.08$ (red), $-0.07$ (green) and $-0.06$ K (blue). The magnetic entropy values are $S/R = 1$ and 2 for solid and dotted lines, respectively. The peak in the isentrope structure observed for the original parameters (red) quickly vanishes upon reducing the magnitude of $J_2$ and hence weakening the frustration. As the strength of the $J_2$ coupling (between the spins on the ring and the central spin) is decreased, even at $J_2 = -0.06$ K, the isentropes begin to resemble those of spin rings (see Supplementary Reference 2).

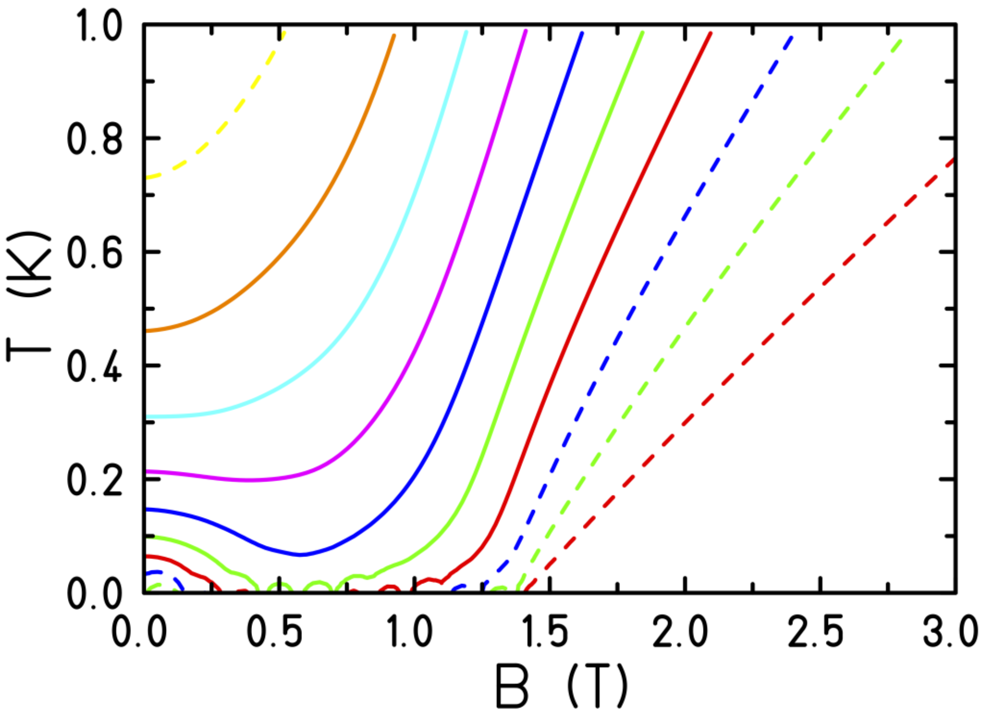


**Supplementary Figure 6. Theoretically calculated isentropes for an equilateral triangle of three spins *s* = 7/2 ($Gd_3$) with exchange *J* = $J_1$ and *g* = 2.02**. Unlike $Gd_7$, the equilateral triangle has a simple level structure leading to a regular staircase profile for the zero-temperature magnetisation (the successive ground-state level crossings give rise to the small bumps in the isentropes close to $T = 0$) and there are no plateaus prior to saturation. Hence, although the triangle is the major motif for many frustrated spin systems, including the triangular or kagome lattice antiferromagnets, its frustration expresses itself quite differently (see Supplementary Reference 3). The $Gd_3$ isentropes (and other thermodynamic properties) are more reminiscent of those of spin rings, which is understandable because the triangle can be considered the smallest spin ring.

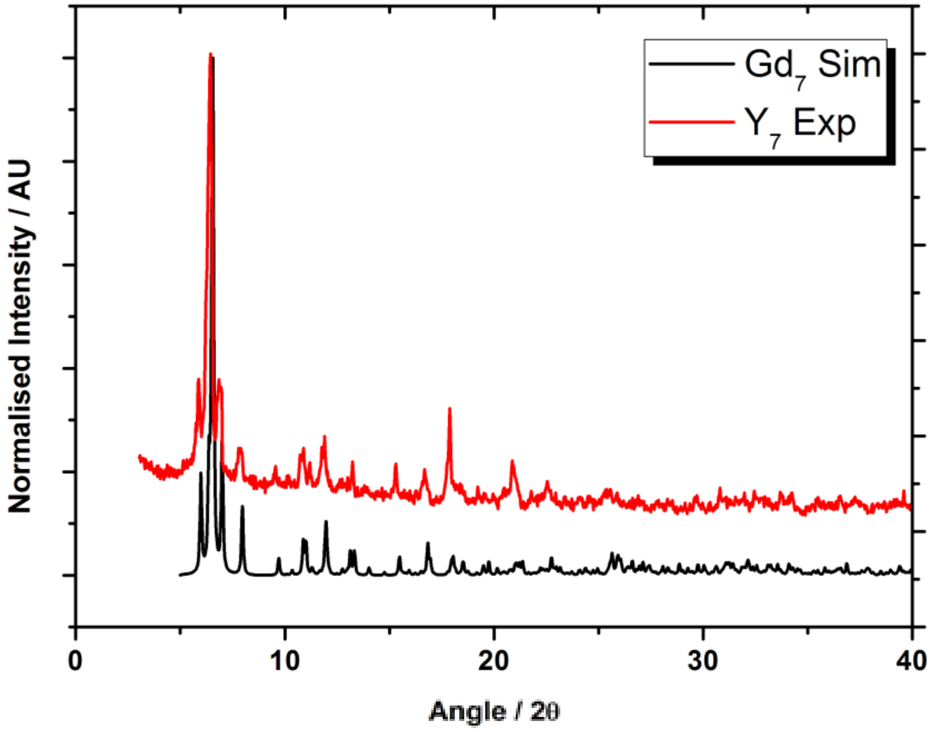


**Supplementary Figure 7**. **Powder X-ray diffraction data for $Y_7$ with that calculated from single crystal data of $Gd_7$.** Unit cell of $Y_7$: $a$ = 16.2134(6), $b$ = 16.4619(6), $c$ = 17.2292(6) Å, $\alpha$ = 97.183(3), $\beta$ = 113.929(3), $\gamma$ = 115.067(3)°, $V$ = 3554.2 Å$^3$. Unit cell of $Gd_7$: $a$ = 16.991(8), $b$ = 16.3943(8), $c$ = 17.2829(9) Å, $\alpha$ = 97.2230(10), $\beta$ = 113.8890(10), $\gamma$ = 114.9720(10)°, $V$ = 3551.81 Å$^3$. See Supplementary Reference 4 for further details.

**Supplementary Note 1. Direct MCE Measurements**

Direct MCE measurements of a pressed pellet sample were performed in a commercial $^3$He setup. The sample-holder consisted of a sapphire plate, with a resistance thermometer (Cernox CX-1010). Wires provided electrical connection, mechanical support and thermal contact to a controlled thermal bath at constant temperature $T_0$. Each MCE measurement started with the sample at zero applied magnetic field $B$ = 0 and $T_0$, and comprised the following four steps: (a) gradual application of a magnetic field, up to a maximum $B_0$; (b) relaxation until the sample reached the thermal equilibrium with the bath; (c) gradual demagnetization down to $B$ = 0; (d) relaxation at zero field until the sample reached thermal equilibrium at $T_0$. Supplementary Figure 2 depicts a representative magnetization-demagnetization cycle, which we experimentally collected for $T_0$ = 0.50 K and $B_0$ = 2 T. During the whole procedure, the as-measured temperature $T$ and applied magnetic field $B$ were recorded continuously.

In order to cope with the unavoidable lack of adiabatic conditions, we related the as-measured $T$ to the adiabatic temperature $T_{ad}$, by evaluating the experimental entropy gains (losses) of the sample due to heat dissipated from (to) the thermal bath. Note that the entropy change of the sample in a time interval, $t - t_0$, is

$$\Delta S = \int_{t_0}^{t} \frac{\kappa(T_0 - T)}{T} \mathrm{d}t, \tag{1}$$

where $\kappa$ is the thermal conductance of the wires, which was previously measured as a function of the temperature, using a free-oxygen copper block as the sample. Since the entropy is related to the heat capacity, $C$, by $S = \int (C/T)\mathrm{d}T$ for a constant applied field, deviations of the as-measured temperature from the temperature, $T_{ad}$, of the corresponding ideal adiabatic procedure

yield

$$\Delta S = \int_{T_{\mathrm{ad}}}^{T} \frac{C}{T}\,\mathrm{d}T. \tag{2}$$

Therefore, the equivalency between Supplementary Eq. (1) and Eq. (2) can be used to straightforwardly obtain $T_{\mathrm{ad}}$ at every time, knowing $\kappa$ and $C$. See, for example, the so-obtained $T_{\mathrm{ad}}(t)$ for the full magnetization-demagnetization cycle (*e.g.*, starting from $t_0 = 0$), depicted in the Supplementary Figure 2. Note that $T_{\mathrm{ad}}$ reverts to its initial value (0.50 K) at the end of the cycle, as expected under adiabatic conditions, thus proving the validity of the post-acquisition data procedure.

Supplementary Figure 3 shows the time-evolution of the temperature data for the demagnetization half-cycle, which starts at $t_0 \approx 1330$ s in the Supplementary Figure 2. In this case, the adiabatic correction to the as-measured temperature is applied limitedly to the time interval corresponding to the applied field change from 2 T to 0, for $T_0 = 0.50$ K.

Finally, Supplementary Figure 4 (and Figure 4 of the main text, only for sub-Kelvin temperatures) reports the so-obtained field-dependent $T_{\mathrm{ad}}$ data, in the form of isentropes, as obtained on magnetization (*e.g.*, $20 < t < 120$ s, in Supplementary Figure 2) and demagnetization (*e.g.*, 1330 s $< t <$ 1530 s, in Supplementary Figure 2).

**Supplementary References**


1. Evangelisti, M. & Brechin, E. K. Recipes for enhanced molecular cooling. *Dalton Trans.* **39**, 4672–4676 (2010).

2. Zhitomirsky, M. E. & Honecker, A. Magnetocaloric effect in one-dimensional antiferromagnets. *J. Stat. Mech.: Theor. Exp*. P07012 (2004).

3. Schnack, J. Effects of frustration on magnetic molecules: a survey from Olivier Kahn until today. *Dalton Trans*. **39**, 4677–4686 (2010).

4. Sharples, J. W., Zheng, Y.-Z., Tuna, F., McInnes, E. J. L. & Collison, D. Lanthanide disks chill well and relax slowly. *Chem. Commun.* **47**, 7650–7652 (2011).
